# Hydrolysis pattern analysis of xylem tissues of woody plants pretreated with hydrogen peroxide and acetic acid: rapid saccharification of softwood for economical bioconversion

**DOI:** 10.1186/s13068-021-01889-y

**Published:** 2021-02-06

**Authors:** Dae-Seok Lee, Yoon-Gyo Lee, Eun Jin Cho, Younho Song, Hyeun-Jong Bae

**Affiliations:** 1 Bio-Energy Research Center, Chonnam National University, Gwangju, 500-757 Republic of Korea; 2 Department of Bioenergy Science and Technology, College of Agriculture and Life Sciences, Chonnam National University, Gwangju, 500-757 Republic of Korea

**Keywords:** Woody plant, Pretreatment, Xylem tissues, Enzymatic hydrolysis, Cellulose recalcitrance

## Abstract

**Background:**

Woody plants with high glucose content are alternative bioresources for the production of biofuels and biochemicals. Various pretreatment methods may be used to reduce the effects of retardation factors such as lignin interference and cellulose structural recalcitrance on the degradation of the lignocellulose material of woody plants.

**Results:**

A hydrogen peroxide-acetic acid (HPAC) pretreatment was used to reduce the lignin content of several types of woody plants, and the effect of the cellulose structural recalcitrance on the enzymatic hydrolysis was analyzed. The cellulose structural recalcitrance and the degradation patterns of the wood fibers in the xylem tissues of *Quercus acutissima* (hardwood) resulted in greater retardation in the enzymatic saccharification than those in the tracheids of *Pinus densiflora* (softwood). In addition to the HPAC pretreatment, the application of supplementary enzymes (7.5 FPU cellulase for 24 h) further increased the hydrolysis rate of *P. densiflora* from 61.42 to 91.94% whereas the same effect was not observed for *Q. acutissima*. It was also observed that endoxylanase synergism significantly affected the hydrolysis of *P. densiflora*. However, this synergistic effect was lower for other supplementary enzymes. The maximum concentration of the reducing sugars produced from 10% softwood was 89.17 g L^−1^ after 36 h of hydrolysis with 15 FPU cellulase and other supplementary enzymes. Approximately 80 mg mL^−1^ of reducing sugars was produced with the addition of 7.5 FPU cellulase and other supplementary enzymes after 36 h, achieving rapid saccharification.

**Conclusion:**

HPAC pretreatment removed the interference of lignin, reduced structural recalcitrance of cellulose in the *P. densiflora*, and enabled rapid saccharification of the woody plants including a high concentration of insoluble substrates with only low amounts of cellulase. HPAC pretreatment may be a viable alternative for the cost-efficient production of biofuels or biochemicals from softwood plant tissues.

## Background

Lignocellulosic biomass is considered as a sustainable and alternative resource with the potential to reduce the dependency on or even replace petroleum for energy production. Woody plants are regarded as economically less feasible than agricultural residues (e.g., rice, wheat, and rapeseed straw) or biological waste materials (e.g., fruit peels, vegetables, and food ingredients) for bioenergy, production due to degradation difficulties associated with the structural and chemical properties of plant cell walls. Despite these difficulties, woody biomass may still be considered an attractive and renewable feedstock due to its high glucan content and abundance in nature [1].

Cell walls of woody plants consist of lignin, hemicellulose, and cellulose. Lignin is connected to hemicellulose, and coats microfibril units (ranging from 10 to 35 nm in diameter) including several elementary fibril groups comprised of β-1,4-glucose chain bundles [2–4]. These microfibrils aggregate to form macrofibril structures (whose sizes range, e.g., between 0.5 and 2 μm for softwood kraft pulp), which in turn assemble to form primary and secondary tracheid cell walls and wood fibers in the xylem tissues of softwood or hardwood [4]. This structural complexity of lignocellulosic biomass hinders enzymatic degradation performed by microbes or fungi. Therefore, various pretreatment methods involving the usage of dilute acids, steam, organosolv, or sodium sulfite have been developed in the past to improve the efficiency of enzymatic saccharification for lignocellulose degradation.

The molecular mechanism of enzymatic hydrolysis of softwood kraft pulp fibers and the relationship between fiber fragmentation and dislocation sites have been evaluated previously [5]. Supramolecular interactions at cellulase dislocation sites for softwood tracheids have also been found [6, 7]. In addition, the cellulose binding module CBM44 (high affinity to the amorphous region of cellulose) was found to bind to the dislocation sites of hardwood-derived dissolving pulp at a greater level than CBM2a (high affinity to crystalline cellulose). Furthermore, fiber fragmentation level with endoglucanase Cel5A was higher than those with xylanase XYN10A, the cellobiohydrolase Cel7A, and swollenin [3].

Hardwoods or softwoods are two types of woody plants. In the context of biofuel production from lignocellulosic biomass, pine trees and poplar are representative softwood and hardwood species, respectively. Hardwoods are composed of wood fibers, tracheids, ray parenchyma cells, and vessel elements in the sap and heart wood [8], whereas softwoods are comprised of tracheids, ray parenchyma cells, and resin canals. Wood fibers (62.4%) and tracheids (91.8%) are the major components of hardwood (e.g., oak) and softwood (especially pine trees), respectively [9]. These xylem components also constitute major physical limitations that reduce hydrolysis efficiency. Analysis of tracheid and wood fiber hydrolysis patterns at both micro and macromolecular levels is therefore required for a better understanding of the lignocellulose bioconversion processes.

The efficiency of enzymatic hydrolysis of woody plants depends on the choice of the pretreatment method and its severity. For example, steam explosion was shown to increase the effectiveness of saccharification of corn stover more than those of poplar and lodgepole pine, whereas the organosolv technique showed higher saccharification efficiency with poplar than with pine or corn stover [10]. Dilute acid was found to be more effective for poplar and eucalyptus rather than spruce [1]. Sulfite pretreatment also led to over 90% saccharification with both soft and hardwoods. Hence, enzymatic hydrolysis efficiency clearly depends on the pretreatment method as well as lignocellulosic biomass type.

Three properties or components of wood materials act as “retardation factors” that limit or determine pretreatment and saccharification efficiencies: (1) the cuticle and epicuticular waxes on plant epidermal tissues, such as those found in corn stover, wheat, and rice straws, (2) lignification degrees of xylem tissues, (3) the structural heterogeneity and complexity of cell-wall constituents such as microfibrils and matrix polymers [11]. The delignification and mechanical delamination of lignocellulosic biomass were shown to be useful methods to study structural recalcitrance of lignocellulosic biomass at micro- and macrofibril levels [12, 13]. Hydrogen peroxide-acetic acid (HPAC) pretreatment was previously reported to reduce lignin content of rice straw, pine wood, and oak wood by 85.12%, 98.08% and 97.61%, respectively [12]. Hence, delignified rice straw was shown to yield lower hydrolysis efficiency than delignified pine or oak wood. Considering the presence of cuticle and epicuticular waxes on epidermal tissues of rice straw, these components may negatively affect the rice straw delignification process and limit the efficiency of cellulase. Further comparisons of enzymatic hydrolysis patterns of HPAC-delignified hardwoods and softwoods can be performed via assessments of the structural heterogeneity and complexity of cell-wall constituents.

Here, hydrolysis pattern of xylem tissues from HPAC-pretreated hard- and softwoods of various wood plant types and cellulose recalcitrance in tracheids and wood fiber of these materials were analyzed to assist the development of rapid hydrolysis methods for economically feasible production of biofuels.

## Results and discussion

### Change in recalcitrance during enzymatic hydrolysis

Various pretreatment methods such as dilute acid addition, steam explosion, organosolv, and sulfite pretreatment have been used to improve enzymatic hydrolysis efficiency of woody biomass [1]. Steam explosion was found to be more advantageous for enzymatic hydrolysis of hardwoods rather than softwoods with reported conversion rates of 65–83% (hardwoods) and 21–69% (softwoods). Similar to steam explosion, dilute acid pretreatment produced readily hydrolysable cellulose fibers from hardwoods, and was shown to yield enzymatic conversion rates of around 80% for hardwood eucalyptus [14], and 40% and 20–70% for softwood spruce and red pine, respectively [1, 15]. Organosolv and sulfite pretreatments have been reported to achieve high conversion rates (over 90%) for both hardwoods and softwoods [1, 16]. These results suggest that each pretreatment method is suitable for a particular type of lignocellulosic biomass in terms of reducing the lignin interference and the structural recalcitrance of cellulose. Therefore, these methods should be selectively utilized to enhance enzymatic saccharification of woody plants.

**Fig. 1:**
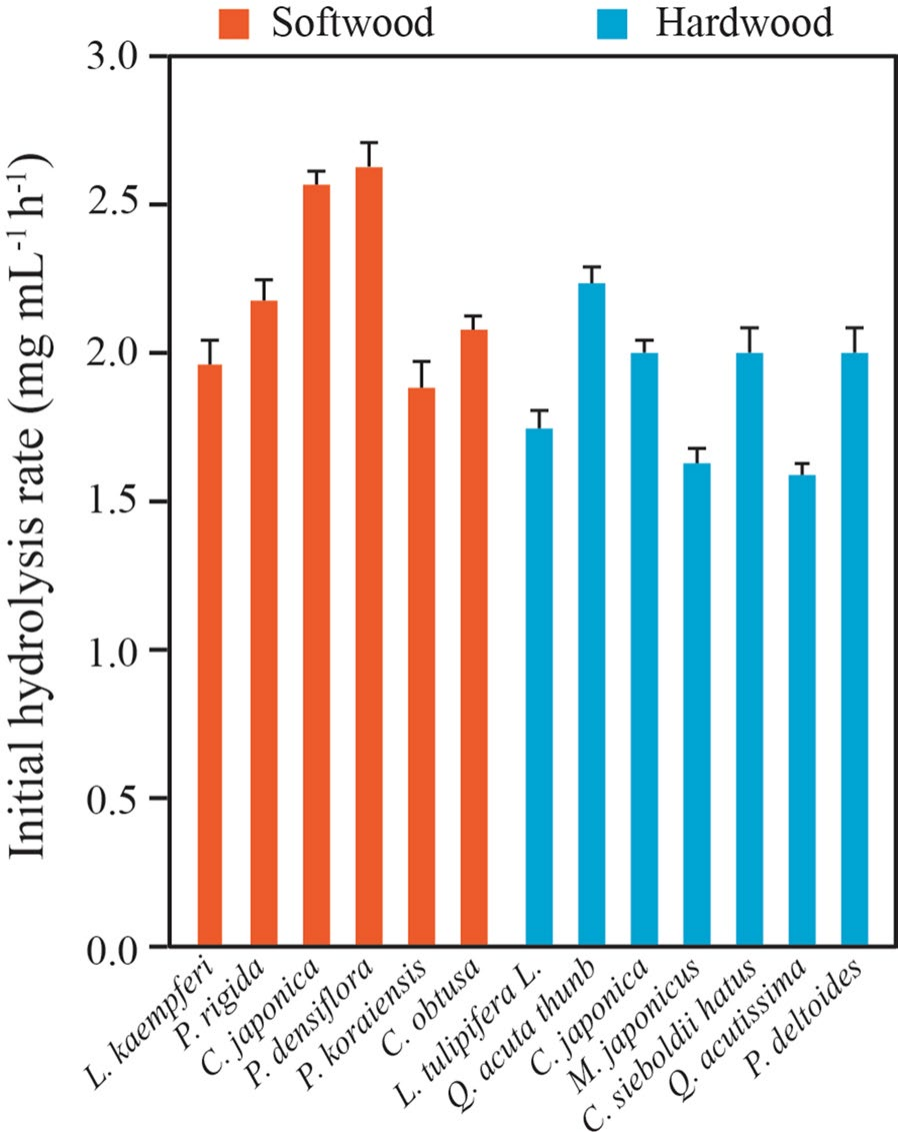
Comparison of initial hydrolysis rate of various woody plants. The HPAC-pretreated substrates (1%, w/v) were hydrolyzed using 7.5 FPU cellulase g biomass ^−1^ at 50 °C for 3 h. The initial rate of *P. densiflora* showed statistically significant difference to the rate of *M. japonicus, Q. acutissima*, and *P. deltoides* (*P* = *0.029*); *C. japonica* presented a statistically significant difference to the rate of *Q. acutissima (P* =  < *0.001)*

HPAC pretreatment has been found to delignify woody substrates efficiently, as evidenced by 98.08% and 97.61% reduction in acid-insoluble lignin content from pine and oak, respectively. Moreover, swelling of xylem tissues has been observed as well [12]. Substrate concentration affects the enzymatic conversion rate and greatly contributes to end-product inhibition and cellulose recalcitrance. Here, initial hydrolysis rates of HPAC-pretreated softwoods and hardwoods were investigated at low substrate concentration to minimize end-product inhibition. HPAC pretreatment was performed on several softwoods (*Larix kaempferi, Pinus rigida, Cryptomeria japonica, Pinus densiflora, Pinus koraiensis,* and *Chamaecyparis obtuse*) and hardwoods (*Liriodendron tulipifera L., Quercus acuta* Thunb.*, Camellia japonica, Mallotus japonica, Castanopsis sieboldii* Hatus*, Quercus acutissima,* and *Populus deltoides*), followed by hydrolysis using 7.5 FPU cellulase at 50 °C for 3 h. Finally, the initial rates were calculated (Fig. 1). In contrast to previous results from steam explosion and dilute acid pretreatments [1], HPAC-pretreated softwoods hydrolyzed faster than the hardwoods. This was especially evident from data for *P. densiflora and C. japonica.* Q*.* Thunb and *Q. acutissima* yielded the highest and lowest hydrolysis rates among all hardwoods, respectively. *P. densiflora* (softwood) and *Q. acutissima* (hardwood) showed highly different hydrolysis rates, and were thus included in further experiments to evaluate rate–limiting factors related to the structural recalcitrance of cellulose at both macro- and microfibril levels during enzymatic hydrolysis. *P. densiflora and Q. acutissima* are also representative softwood and hardwood species from forests in Korea, respectively.

### Analysis of structural recalcitrance

The variation in the initial hydrolysis rates implies variation in the structural recalcitrance of wood plants as well. Hydrolysis was therefore repeated multiple times (re-hydrolysis) for *P. densiflora* and *Q. acutissima* to further study the variation in the initial hydrolysis rates. One round of re-hydrolysis included the hydrolysis followed by washing. Re-hydrolysis was carried out several times until the reducing sugars released from the substrates were no longer detected.

**Fig. 2:**
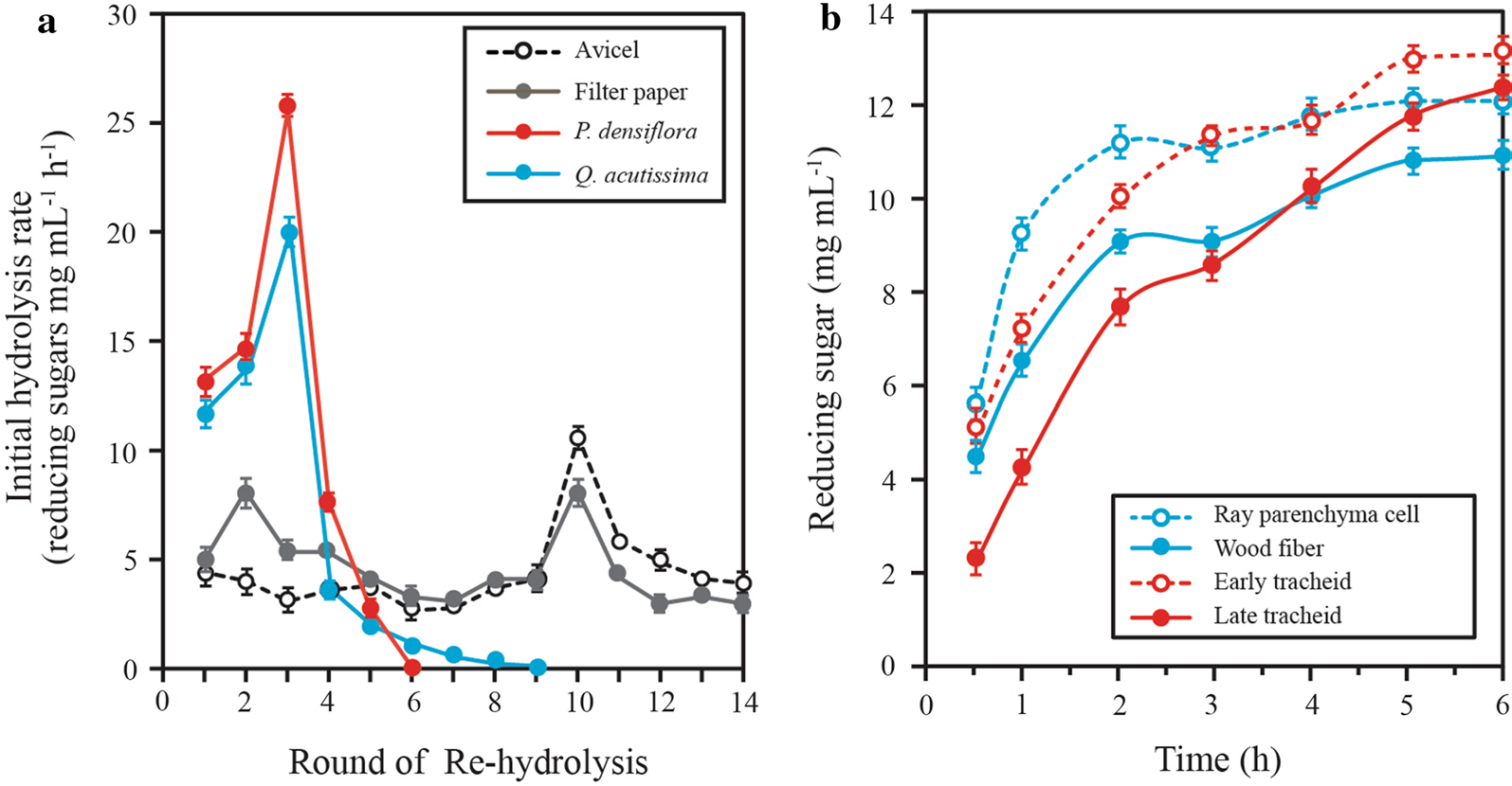
Comparison of the change in recalcitrance during enzymatic hydrolysis. **a** Each re-hydrolysis round consisted of hydrolysis with 7.5 FPU cellulase at 50 °C for 1 h followed by three washing rounds with distilled water. The hydrolysis of *P. densiflora* was complete at the end of 6th round, and the hydrolysis of *Q. acutissima* was complete at the end of the 9th round. Filter paper and Avicel released reducing sugars at the end of the 14th round. **b** The enzymatic degradation of wood fibers and ray parenchyma cells from *Q. acutissima* and tracheids (early and late tracheids) from *P. densiflora* are compared

The initial hydrolysis rate of HPAC-pretreated substrates showed an overall steep increase until the third re-hydrolysis round. This indicates that as hydrolysis proceeded, the effect of structural recalcitrance become apparent, particularly in comparison to commercial substrates such as filter paper or Avicel (Fig. 2a). This result is also consistent with a previous report on the gradual increase in crystallinity of pre-hydrolyzed lodgepole pine [17].

A relative proportions of the I_α_ and I_β_ crystalline forms (CrI, cellulose crystallinity indices) of cellulose was estimated to distinguish higher ratios for softwood than for hardwood [18, 19]. Natural crystalline cellulose (cellulose I) is convertible to more readily hydrolysable cellulose II or III (hydrolysis rate: cellulose I < cellulose II < cellulose III) by pre- or post-treatment [20, 21]. The structural modification of the crystalline form from cellulose I_α_ to cellulose III_1_ induced greater adsorption of Cel7A in the cellulose hydrophobic region, and accelerated the hydrolysis of the crystalline cellulose [22]. Morphology change of wood fiber of hardwood by HPAC pretreatment was observed as more cellulase-friendly cellulose (cellulose II or III) causing remarkable cellulose swelling in the lumen site of the wood fiber compared to acidified sodium chlorite (ASC) pretreatment, which was sorted into readily hydrolysable (72.9%), mid-hydrolysable (8.2%), and hardly hydrolysable (18.9%) cellulose forms [23]. In Fig. 2, the hydrolysis of *P. densiflora* was completed at the end of the 6th round, whereas that of *Q. acutissima* proceeded until the 9th round, and undigested solid cellulose residues were present even after 14th round for Avicel and filter paper. These results indicate that structural properties of cellulose from each biomass type determine the enzymatic hydrolysis rate and hardly hydrolysable cellulose was less remained in *P. densiflora* than *Q. qcutissima* under HPAC pretreatment condition.

The carbohydrate composition and surfaces characterization of tracheids and wood fibers obtained from softwood and hardwoods were investigated through binding affinity of fluorescent-tagged carbohydrate-binding modules [24]. Xylem tissues, including tracheids, ray parenchyma cells, and wood fibers of different softwood and hardwood types, have been predicted to form different lignin content, carbohydrate composition, and cellulose structures, which affects the degrees of resistance to cellulases that retard saccharification to varying degrees. However, this hypothesis has not yet been examined. The analysis of xylem tissues may enable an estimation of the structural effect of cellulose on enzymatic hydrolysis, and therefore, ray parenchyma cells, wood fibers, and tracheids from *P. densiflora* and *Q. acutissima* were isolated and their hydrolysis rates were determined. Tracheids of *P. densiflora* were separated into either early or late tracheids, where the xylem tissues of *Q. acutissima* were separated into ray parenchyma cells and wood fibers (including medullary rays and fiber tracheids). Figure 2b indicates that ray parenchyma cells yielded the highest hydrolysis rates in the early stages of hydrolysis, and the concentration of reducing sugars later approached a steady level. No further increase in reducing sugar concentration was detected when the incubation time was prolonged to 24 h. Late tracheids and wood fiber showed the strongest recalcitrance at the early stage and late stages (after 4 h) of hydrolysis, respectively.

### Softwood and hardwood degradation patterns

Fiber cutting (fragmentation) mechanism can be observed at the macromolecular level during the enzymatic hydrolysis of lignocellulosic biomass [5, 7]. Fiber cutting was found to level off with shorter fiber lengths between 130 and 220 μm during the early stage, the length of which varies depending on pretreatment conditions and the chemical composition of the substrate [25].

**Fig. 3:**
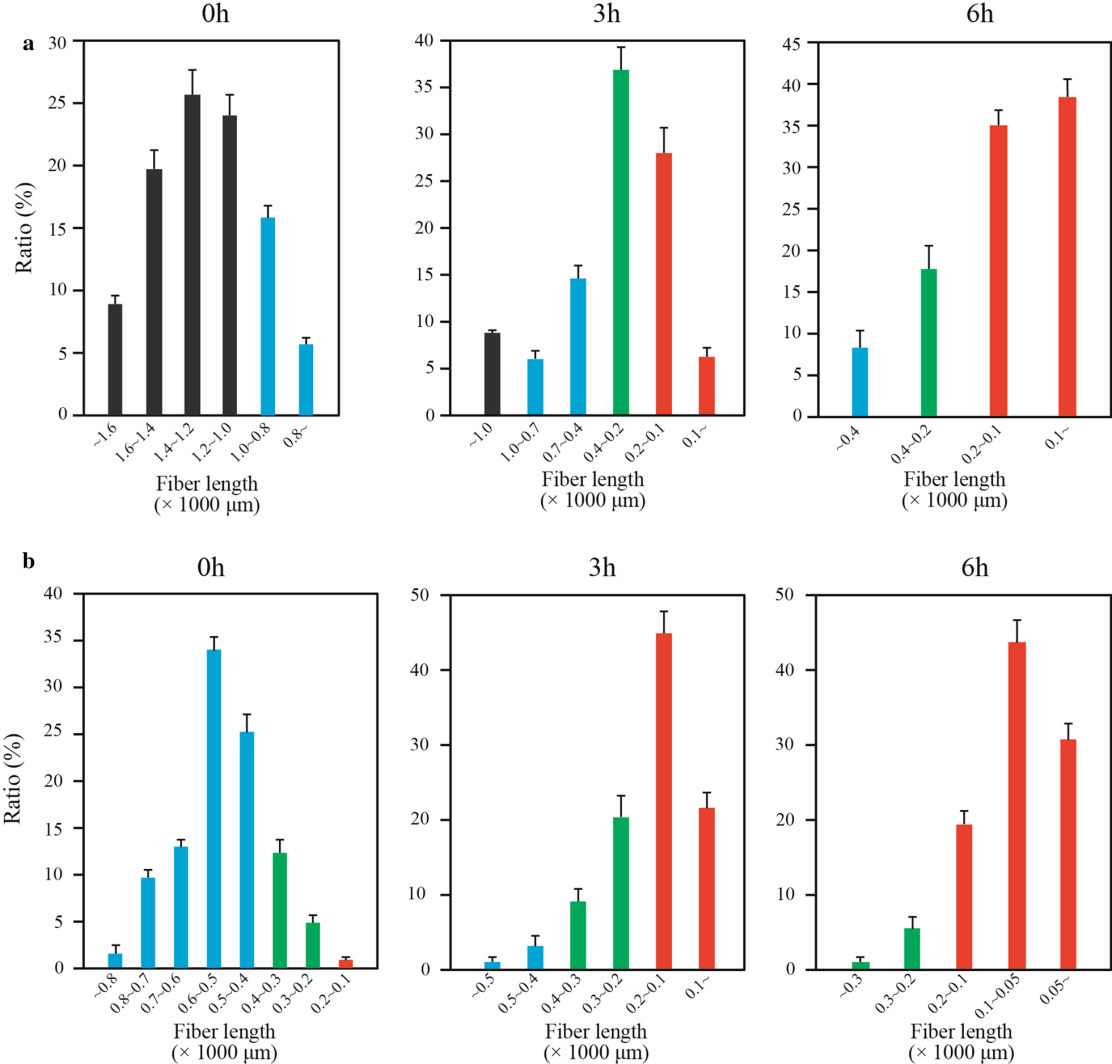
Fiber cutting pattern during enzymatic hydrolysis. **a** The fragmentation of *P. densiflora* (0 h, 3 h, and 6 h) and **b** *Q. acutissima* (0 h, 3 h, and 6 h). Black color, > 1000 μm; blue color, 400 ~ 1000 μm; green color, 200 ~ 400 μm; orange color, $<$ 200 μm

Here, *P. densiflora* and *Q. acutissima* were subjected to HPAC pretreatment and subsequent hydrolysis to investigate fiber cutting mechanism (Additional file 1: Figure S1). The initial average length of the tracheids from *P. densiflora* was 1239.06 ± 301.68 μm (Fig. 3), which includes tracheids fragmented during pretreatment or preparation. The lengths of major tracheid fragments ranged between 900 and 1600 μm. Initial fiber fragments (ranging between 500 and 2100 μm in length) were enzymatically hydrolyzed for 3 or 6 h. The ratios of the length of resulting fragments with respect to the average length ranged between 1/4 and 1/8, and the amount of these fragments accounted for 64.63% of all fragments after 3 h hydrolysis. After 6 h, this ration ranged between 1/8 and 1/20, and fragments within this size range accounted for 73.53% of all fragments. For *Q. acutissima*, the initial average length of wood fibers was 515.9 μm, and lengths ranged between 200 and 850 μm. The ratios of lengths of resulting fragments with respect to the average length ranged between 1/4 and 1/8 as well, and the amount of these fragments accounted for 66.55% of all fragments after 3 h. Similar to values found for *P. densiflora*, this ration ranged between 1/8 and 1/20 after 6 h. and fragments within this size range accounted for 74.32% of all fragments.

**Fig. 4:**
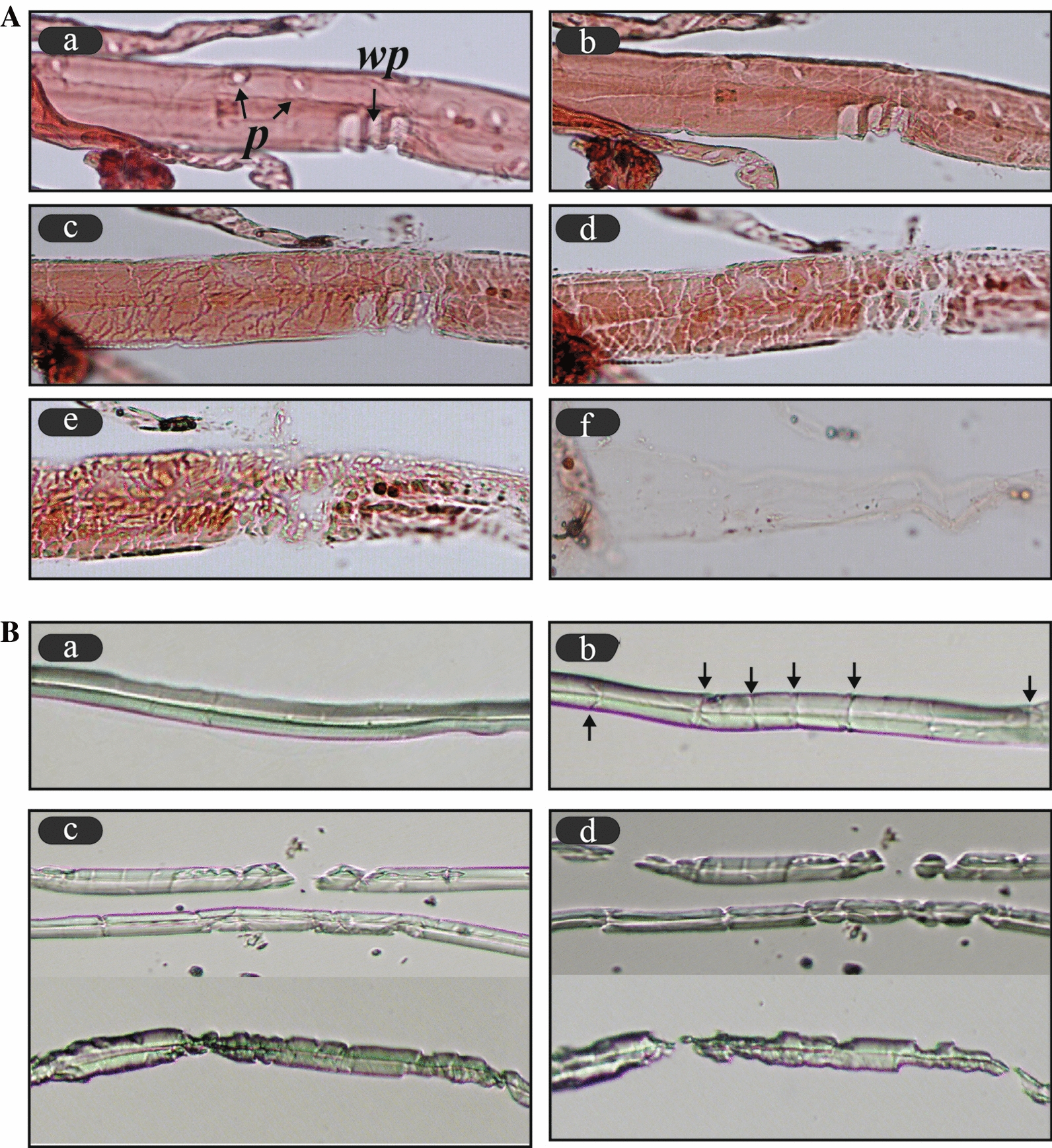
Comparison of softwood and hardwood hydrolysis patterns. **A** Softwood tracheid degradation patterns. (a) 0 h; (b) 0.5 h; (c) 1 h; (d) 2 h; (e) 6 h; (f) 24 h. *p*: pit; *wp*: window-like pit. **B** Hardwood wood fiber degradation. a, 0 h; b, 0.5 h; c, 2 h, d, 3 h. Arrows denote the cracks generated by the enzymes

The fiber cutting and fragmentation patterns were thus insufficient to explain why faster overall hydrolysis rates were observed for *P. densiflora* than *Q. acutissima*. Therefore, tracheids and wood fibers of *P. densiflora* and *Q. acutissima* were comparatively evaluated. For this purpose, the surface of the fragments was monitored for 24 h after cellulase treatment (Fig. 4). The widths of tracheids ranged between 20 and 40 μm, and cell walls included pits and window-like pits. The diameter of lumen of *P. densiflora* was wider than that of wood fibers from *Q. acutissima.* Cellulase- tracheid binding profiles in softwood were previously shown to include high levels endoglucanase (EGV from *Humicola insolens*, GH 45) and cellobiohydrolase (CBH1 from *T. reesei*, GH 7) in the lumen [6]. These characteristics of tracheids allow cellulase to easily approach cellulose fibers on the surface and lumen. Window-like pits were found to be targets for cellulase attacks during the initial stage. The tracheid bodies also cracked and spread out in the form of irregular mosaic shapes, whereas the wood fibers of *Q. acutissima* were longitudinally cracked by cellulase. Deconstruction of the fiber cutting ends and a peeling- or erosion-like effect on the surface of the wood fibers were also observed. The shortened fibers remained intact during the prolonged 24-h incubation. The remaining fragments at the late stage included more recalcitrant cellulose fibers that were resisted cellulase action, implying that the primary wall or S_1_ layer of wood fiber was responsible for this result [23]. These results may thus explain the retardation of *Q. acutissima* degradation compared to that of *P. densiflora*.

### Rapid hydrolyzation of softwood

The reduction of costs associated with pretreatment and enzyme supply has been a major challenge in bioethanol production from biomass. The costs of chemicals, enzymes, and the electricity used in bioethanol production from woody plants has been reported [26]. A study found that the cost of hydrogen peroxide and acetic acid individually accounted for 35.71% of the total cost, and the electricity cost incurred during enzymatic hydrolysis accounted for 83% of the total electricity cost for the entire bioethanol production [26]. These results were based on bioethanol productivity from hardwood. The results of the present study suggest that the enzymatic hydrolysis of hardwood efficiency was lower than that of softwood, due to a barely hydrolysable cellulose being obtained at the latter stage of enzymatic hydrolysis [23]. It indicates that the high efficiency of saccharification and fermentation with softwood leads to reduced costs of chemicals and enzymes. There are three methods for reducing cost: the first is an alteration of the chemical ratio such as that of hydrogen peroxide and acetic acid in the pretreatment process, which leads to approximately 88.9% of efficiency under optimum condition [12], thereby realizing a better recycling efficiency of acetic acid. Rapid saccharification with low amount of cellulase is likely to provide the second and third solutions, by increasing enzyme recycling efficiency and reducing electricity consumption, respectively.

There are numerous of factors that affect enzymatic hydrolysis of lignocellulosic biomass such as biomass particle size and fibrillation, pretreatment severity, enzyme source and doze, reaction time. In particular, both weak and strong compatibility among pretreatment methods and biomass feedstocks have been reported. Popping pretreatment showed a high efficiency with the agricultural residues such as rice and rapeseed straw [27–29], but not with woody plant [23]. The hydrolysis efficiencies of eucalyptus and cedar that were pretreated with NaOH were found to be approximately 70% and 80% in 24 h, respectively [30]. Spruce that was pretreated via steam explosion was found to be hydrolyzed at 29% efficiency at 96 h [31]. Dilute acid pretreatment was also shown to increase enzymatic hydrolysis efficiency in hardwoods [1, 14, 15]. Organosolv-pretreated poplar cellulose was found to be converted to glucose at approximately 85% yield for 48 h, and the cellulose of mixed softwoods (spruce, pine, and Douglas fir) showed conversion efficiencies of around 98% [32, 33]. Here, HPAC-pretreated softwood *P. densiflora* and hardwood *Q. acutissima* were rapidly hydrolyzed at low substrate concentrations (1%), resulting in a yield of 90 ~ 100% at 12 h depending on the dosage of cellulase (7.5–30 FPU, data not shown). Hydrolysis patterns of these substrates at the macromolecular level were also different from each other. Based on the results, *P. densiflora* was chosen to achieve rapid saccharification at a highly insoluble substrate, instead of *Q. acutissima* which showed greater recalcitrance during the late stage of hydrolysis [23].

**Fig. 5:**
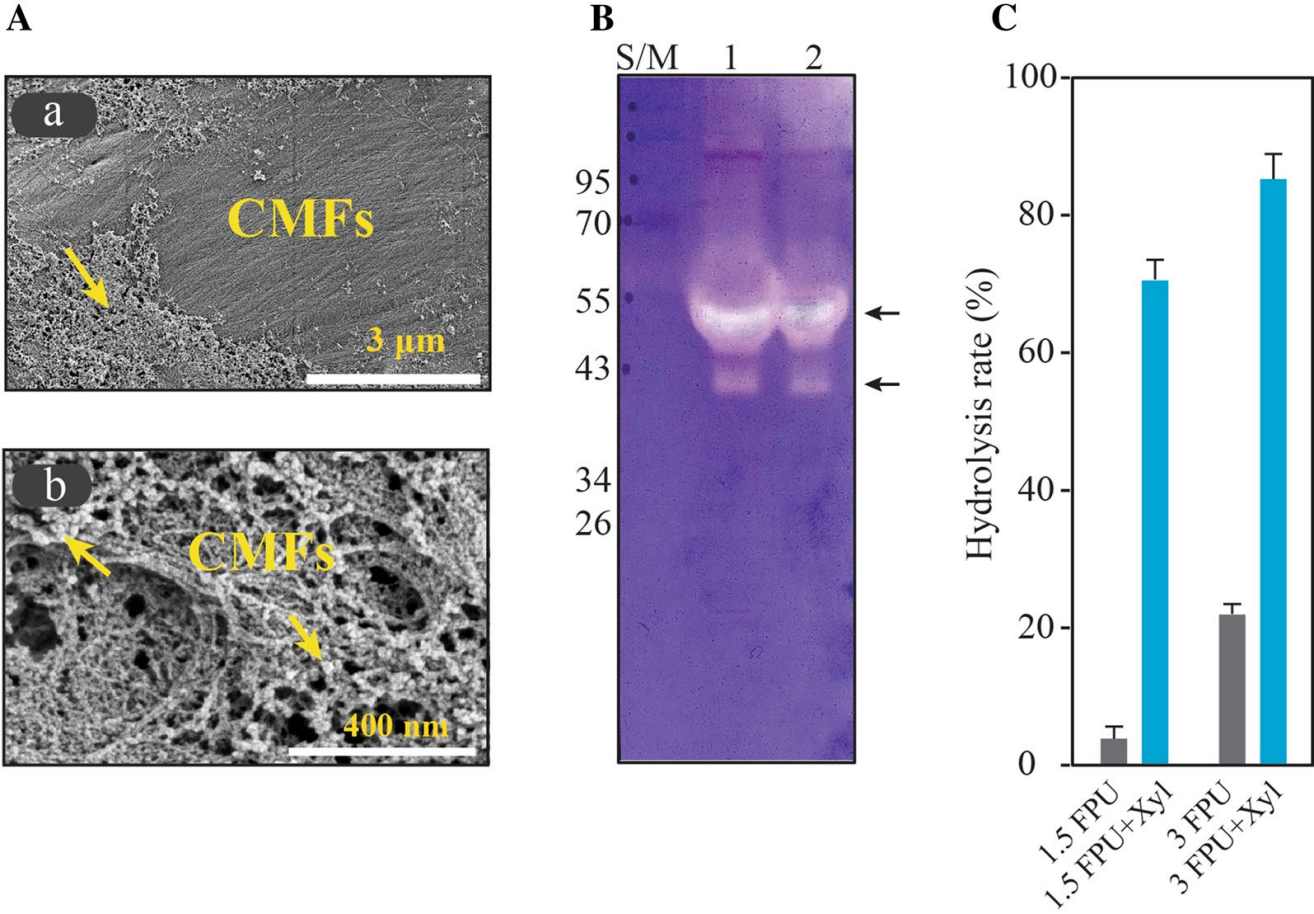
Analysis of synergistic effect of xylanase on HPAC-pretreated *P. densiflora*. **A** Microscope images of the microfibrils of the HPAC-pretreated softwood. CMFs, cellulose microfibrils; arrow, hemicellulose or unspecified material (**a** scale bar = 3 μm; **b** scale bar = 400 nm). **B** Zymogram of the enzymes produced by *T. reesei* RUT-C30. Two bands representing mannanase activities in enzyme solution is visible on SDS-PAGE gel (arrows). The enzyme was loaded at 6 μg on lane 1 and 3 μg on lane 2, respectively. (C) Synergistic effect of xylanase in the hydrolysis of 1% HPAC-pretreated *P. densiflora* with 3 FPU for 3 h

The hemicellulose network within softwood is known to hinder the access of cellulase to cellulose fibers during enzymatic hydrolysis. The two main components of softwood hemicellulose are galactoglucomannan and arabinoglucuronoxylan [34]. The chemical composition of hemicellulose of *Pinus radiata* was reported to include xylose at 19% and mannose at 37% [35]. In contrast, Korean red pine (*P. densiflora*) trunk was found to include cellulose, xylan and galactomannan at rates of 41.9%, 6.4%, and 14.9%, respectively [36]. To investigate the possible utility of xylanase addition to HPAC-pretreated *P. densiflora*, microfibril surfaces of tracheids were examined, and found to be covered by hemicellulose or other unspecified materials (Fig. 5).

Extracellular enzymes of *T. reesei* are known to include 68–78% cellobiohydrolases, 10–15% endoglucanases and others enzymes such as beta-glucosidase, xylanases, and accessory enzymes [37–39]. *T. reesei* produces XYN I and XYN II as two main xylanases types in addition to XYN III, and XYN IV. However, zymogram analysis showed that a single 55 kDa band corresponding to XYN IV showed enzymatic activity on birchwood and beechwood xylans [29]. Tenkanen et al. (2012) previously reported that XYN IV showed a typical exo-action on linear β-1,4-xylooligosaccharides [40], and low activity on xylo-biose, -triose, and -tetraose. Xylooligomers were also reported to strongly inhibit the activity of Cel7A (cellobiohydrolase I) by binding to the active site of this enzyme [41, 42]. Moreover, the synergistic action of xylanase and mannanase was shown to improve the total hydrolysis efficiency of softwoods [43]. Here, mannanase activity was also observed in enzymes produced by *T. reesei* RUT C30 (Fig. 5B). Supplementation with xylanase reduced enzymatic inhibition and physical hindrance on microfibril surfaces during hydrolysis of the HPAC-pretreated *P. densiflora*. Addition of xylanase into 3 FPU cellulase increased the hydrolysis efficiency of 1% HPAC-pretreated *P. densiflora,* from 22 to 85% for 3 h and complete hydrolysis was observed at 9 h (Fig. 5C).

**Fig. 6:**
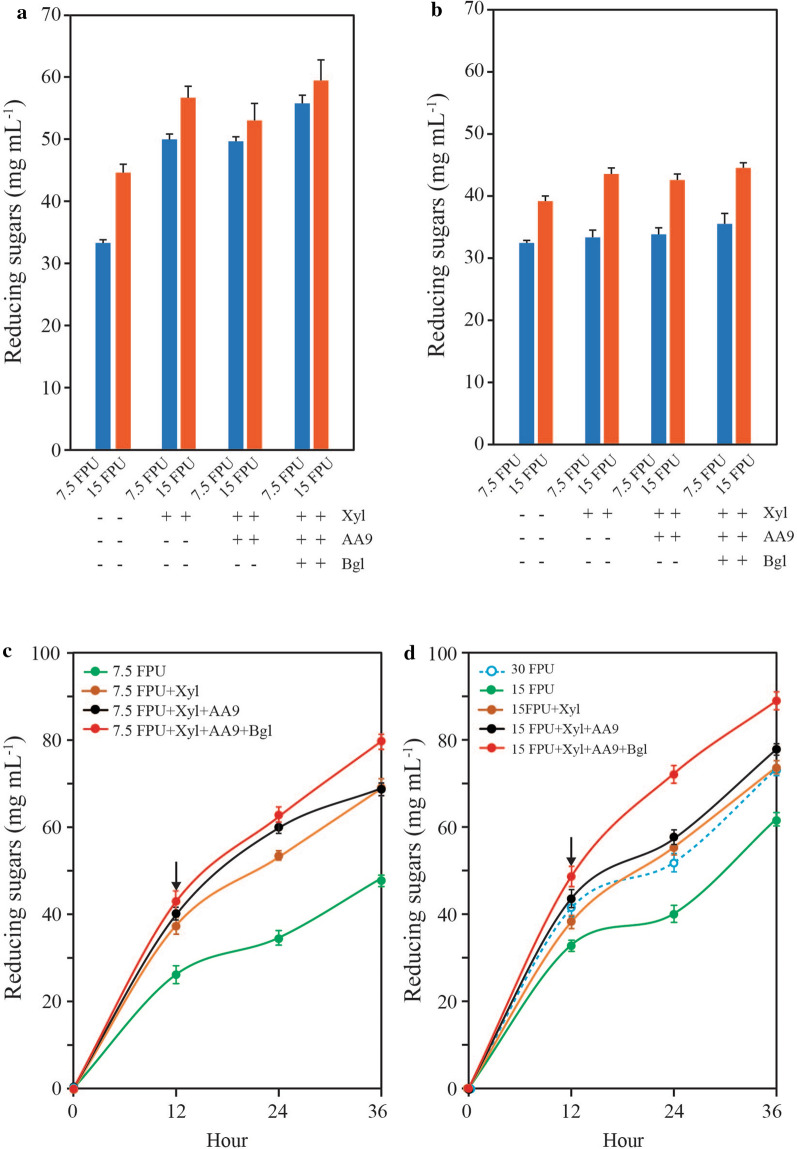
Improvement of the enzymatic hydrolysis rate. **a** 5% HPAC-pretreated *P. densiflora* and **b** 5% *Q. acutissima* were hydrolyzed with 7.5 and 15 FPU cellulase. The supplementation of accessory enzymes contributed to the remarkable hydrolysis efficiency of the HPAC-pretreated softwood through synergistic effects with 7.5 FPU cellulase. Xyl, xylanase; AA9, auxiliary activity family 9 copper-dependent lytic polysaccharide monooxygenases; Bgl, beta-glucosidase. **c** Fed-batch enzymatic hydrolysis using 7.5 FPU g biomass^−1^ and **d** 15 FPU g biomass^−1^ with accessory enzymes (Xylanase, AA9, and beta-glucosidase). The initial concentration (5%) was hydrolyzed for 12 h, and additional substrate was added to obtain a total concentration of 10%, as indicated by the arrow. The most economically feasible enzyme dose was found to be 7.5 FPU cellulase at 10% of the substrate when incubated with accessory enzymes

High substrate concentrations are required to obtain high concentrations of fermentable sugars, and thus produce bioethanol efficiently. However, hydrolysis of high concentration of insoluble substrates was hindered by end-product inhibition that strongly reduced the hydrolysis rate (Additional file 2: Figure S2). Therefore, supplementary enzymes such as xylanase, AA9, and beta-glucosidase were added to cellulase cocktail solutions (7.5 or 15 FPU cellulase) to reduce end-product inhibition and the structural recalcitrance of the substrates (Fig. 6). Xylanase in combination with 7.5 and 15 FPU cellulase increased 24 h hydrolysis efficiency from 61.42% to 91.94% and 104.41%, respectively. No enhancement was found when AA9 was added, while beta-glucosidase addition led to 102.69% and 109.60% 24 h efficiency with xylanase in 7 and 15 FPU cellulase solution, respectively. Xylooligomers and cellobiose are strong inhibitors to cellulases. The monomers from soluble oligomers were released more by the addition of supplementary enzymes, which led to efficiencies of over 100%. In contrast, the synergistic effect of the supplemented enzymes, especially xylanase, was lower on HPAC-pretreated *Q. acutissima* (Fig. 6b), even though this plant contains a higher proportion of xylose as its main hemicellulose component [12]. Overall, these results indicate that hemicellulose causes a retardation of the enzymatic hydrolysis of HPAC-pretreated softwood, while structural recalcitrance of cellulose primarily delays hardwood hydrolysis.

With concentration of insoluble substrates above 5–10%, the hydrolysis rate is negatively affected by inefficient agitation due to higher viscosity, which acts as a strong retardation factor, and leads to more severe end-product inhibition. Here, the initial concentration of *P. densiflora*, which has an insoluble substrate concentration of 5%, was hydrolyzed using 7.5–30 FPU cellulase and other accessory enzymes such as xylanase, AA9, and beta-glucosidase (Fig. 6). After 12 h, insoluble substrate was added to the reaction mixture to obtain a final insoluble substrate concentration of 10%. The maximum concentration of the reducing sugars was found to be 89.17 g L^−1^ at 36 h with 15 FPU cellulase and accessory enzymes, reaching 74.68% of the theoretical maximum rate. Consequently, an economically feasible hydrolysis process was achieved using 7.5 FPU cellulase and other accessory enzymes, which is a remarkable result compared to that with 30 FPU cellulase only.

In summary, the high costs associated with pretreatment methods and cellulolytic enzyme production is a major hindrance to the delivery of cellulosic biofuels to the market. HPAC pretreatment of softwood efficiently reduced lignin interference and cellulose structural recalcitrance. Only a low amount of cellulase was required for the production of a high concentration of fermentable sugars within a short reaction time. Rapid saccharification provides more number of cycles of cellulase recycling that are significantly shorter, while retaining their activity. HPAC for softwood is therefore an advantageous pretreatment for the economical production of biofuels or biochemicals.

## Conclusions

The structural complexity of woody plants results in recalcitrance and prevents efficient enzymatic hydrolysis, and thereby leads to long reaction times for the production of high amounts of fermentable sugars. The level of recalcitrance throughout enzymatic hydrolysis is determined by pretreatment conditions and biomass type. Here, the HPAC pretreatment facilitated the removal of lignin from both softwood and hardwood biomass types. Moreover, this pretreatment method led to a more efficient reduction of cellulose structural recalcitrance of the softwood *P. densiflora*. Rapid enzymatic conversion was achieved with even low cellulase doses and high concentrations of woody plants. The methods described here may enable the economically feasible production of biofuels and biochemicals.

## Methods

### Enzyme preparation

Cellulase was produced from *Trichoderma reesei* in Czapek dox media (0.2% KH_2_PO_4_, 0.42% (NH_4_)_2_SO_4_, 0.03% MgSO_4,_ 0.03% CaCl_2_, 0.1% peptone, 1% glucose, 0.2% trace element solution (0.1% MnSO_4_, 0.2% CoCl_2_, 0.14% ZnSO_4_, 0.5% FeSO_4_), 1% glucose, 0.02% urea, and 2% Tween 80). A 100 L bioreactor (KobioTech, South Korea) was operated at 400×*g* and 28 ºC for 8 days. Cellulase expression was induced by the addition of 1% (w/v) Avicel. The airflow rate and pH adjustment were auto-regulated to 1 vvm and pH 4.8, respectively.

GtAA9- auxiliary activity family 9 (AA9) copper-dependent lytic polysaccharide monooxygenases (LPMOs)- from *Gloeophyllum trabeum* were cloned and expressed in *Pichia pastoris* with the pPICZαA expression vector (Invitrogen, Carlsbad, CA, USA) [44]. The recombinant enzyme was purified with Ni–NTA column (Qiagen Hilden, Germany), and quantified by a Bradford assay.

Xylanase from *T. longibrachiatum* (Cat. X2629) and beta-glucosidase (AnBgls) from *Aspergillus niger* were purchased from Sigma-Aldrich and Megazyme Inc. (Lot 141001, Bray, Ireland), respectively.

### Comparison of the hydrolysis rate of various lignocellulosic biomasses

Softwoods (*L. kaempferi, P. rigida, C. japonica, P. densiflora, P. koraiensis, and C. obtuse)* and hardwoods (*L. tulipifera* L*., Q. acuta* thumb.*, C. japonica, M. japonicus, and C. sieboldii* Hatus.*, Q. acutissima,* and *P. deltoides*) were pretreated by the HPAC method as follows: The substrates (1%, w/v) were hydrolyzed with 7.5 FPU cellulase in 1 mL of 0.1 M citrate buffer (pH 5.0) at 50 °C for 3 h. The reducing sugars were measured by 3,5-dinitrosalicylic acid (DNS) assays, and the initial rate (mg of reducing sugars per hour) of each substrate was calculated.

### Preparation of delignified fibers and xylem tissues fraction

Pine (*P. densiflora*, diameter: 13 cm) and oak (*Q. acutissima*, diameter: 15 cm) trees were used to prepare the substrates that were used in all enzymatic saccharification processes. The wood was cut to 0.2 cm (width) × 0.3 cm (height) × 4 cm (length) pieces. A hydrogen peroxide (H_2_O_2_; 28% stock solution purchased from Duksan Reagents, Korea) and acetic acid (CH_3_COOH; 99% stock solution purchased from Duksan Reagents, Korea) solution (HPAC) was prepared in a 1:1 ratio and 1 L of the solution was added to 100 g lignocellulosic biomass [12]. The mixture was incubated in a water bath at 80 °C for 2 h and then washed several times to remove the solution. The delignified samples were squeezed and freeze-dried. The substrates were used to conduct enzymatic saccharification and to separate xylem tissues from one another.

Delignified pine and oak wood, Avicel, and filter paper were used to measure change of in the strength of cellulose fiber recalcitrance during enzymatic hydrolysis. Each sample (1%) was hydrolyzed with 10 FPU cellulase g^−1^ biomass in 0.1 mol L^−1^ citrate buffer (pH 0.5) at 50 °C for 1 h. The reducing sugar concentration in the supernatant was measured, and the pellet fraction was washed for subsequent hydrolysis. The pellet was then hydrolyzed under the same conditions as previously described. This was considered to be one subsequent hydrolysis cycle. Six to 14 subsequent hydrolysis cycles were performed depending on the substrate. Recalcitrance of the substrates can be analyzed by measuring the initial rate of each round.

The fibers, tracheids, medullary rays, and ray parenchyma cells of HPAC-pretreated oak were separated by filtration with 60 and 100 mesh (S1020, Sigma-Aldrich). HPAC-pretreated pine tracheids were separated, and the tissue fractions (0.5%, g/v) were hydrolyzed in 0.1 M citrate buffer (pH 5.0) containing 15 FPU cellulase at 50 °C for 6 h. Nelson-Somogyi (NS) and DNS assays were used to measure the concentration of the reducing sugars [29].

### Analysis of wood hydrolysis pattern

Tracheids or wood fibers (2%, g/v) from the pine and oak were hydrolyzed with 7.5 FPU cellulase in 1 mL of 0.1 M citrate buffer (pH 5.0) at 50 °C. The hydrolysis patterns were monitored and the lengths of tracheids or fibers were measured via microscopy (Eclipse TE2000-U, Nikon; Olympus BX41).

Tracheids were stained with Congo-red and were placed on glass slides, a rectangle wall was made with nail enamel, and 0.1 M citrate buffer (pH 5.0) containing 1.5 FPU cellulase was added. The glass slides were sealed with a cover glass and nail enamel and placed in a small batch incubator and incubated at 50 °C. The tracheids were observed for 24 h. The same process was employed for hardwood fibers.

### Scanning electron microscopy (SEM)

The surface morphology of tracheids from HPAC-pretreated pine was observed using a JSM-7500 F (Jeol, Japan) field-emission scanning electron microscope with a beam voltage of 3 kV. Briefly, the sample was dehydrated with a graded ethanol series and freeze-dried. After being sputter-coated with osmium, the external surface of the sample was observed.

### Zymogram analysis of mannanase

The mannanase activity in cellulase produced by *T. reesei* RUT C30 was analyzed using a modified method described by [45]. The cellulase (3, 6 μg) was loaded on SDS-PAGE with galactomannan. After electrophoresis, the gel was incubated in refolding buffer (20 mM citrate buffer pH 5.0, 0.1 mM CaCl_2_, 1% Triton X-100) at room temperature for 30 min, and washed with 20 mM citrate buffer (pH 5.0). The reaction of the enzyme was performed at 37 °C for the appropriate time. The gel was neutralized in phosphate buffer (pH 7.0) and stained with Congo red. Destaining was conducted with 1 M NaCl solution until a white band appeared. Citrate buffer (pH 3 ~ 5) was added to turn the red background to dark blue for better clarity.

### Enzymatic hydrolysis of *P. densiflora*

The saccharification efficiency of different concentrations of pretreated pine was analyzed. One per cent (w/v) of HPAC-pine was hydrolyzed with 7.5 FPU cellulase, 5 units of xylanase, and 10 μg of AnBgls in 1 mL of 0.1 M citrate buffer (pH 5.0) at 50 °C for 24 h. This amount of time was sufficient to degrade the solid fraction of the substrate. The supernatant was subsequently incubated until the cellobiose was completely digested to glucose in the solution. The reducing sugar concentration was measured by DNS assay. A total of 12.01 mg mL^−1^ of reducing sugars from 1% HPAC-pretreated pine was assigned as the standard concentration. The theoretical concentration of reducing sugars from each concentration of HPAC-pine was calculated by multiplying the standard concentration by each concentration. One unit of xylanase was defined as the amount of xylanase that produced 5 mg mL^−1^ of reducing sugars from 1% beechwood xylan during 10 min at 50 °C. A total of 40 μg mL^−1^ AnBgls was determined to be the enzyme amount that completely hydrolyzed 20 mM cellobiose to glucose during 10 min at 50 °C.

To achieve the rapid saccharification of HPAC-pine, 1% (w/v) HPAC-pretreated pine was incubated with 1.5 ~ 3 FPU cellulase or cocktail solution of 1.5 ~ 3 FPU cellulase with 5 units of xylanase (*T. longibrachiatum*, Sigma) in 1 mL of 0.1 M citrate buffer (pH 5.0) at 50 °C for 3 h. The enzymatic hydrolysis of 5 or 10% of HPAC-pretreated pine was also determined in 1 mL of 0.1 M citrate buffer (pH 5.0) containing 7.5 ~ 15 FPU cellulase with or without 5 units of xylanase, 2.4 μg of AA9, and 50 μg of AnBgls at 50 °C for 24 h.

## Supplementary information

**Additional file 1: Figure S1.** The fragmentations of HPAC-pretreated softwood and hardwood. (A) *P. densiflora*; (B) *Q. acutissima*. Tracheids or wood fibers from *P. densiflora* and *Q. acutissima* were hydrolyzed with 7.5 FPU cellulase (50 μL mL^-1^) g biomass^-1^ in 1 mL of 0.1 M citrate buffer (pH 5.0) at 50 °C. a, 0 h; b, 3 h; c, 6 h.

**Additional file 2: Figure S2.** The comparison of end-product inhibition effect on various substrate concentrations of *P. densiflora* during enzymatic hydrolysis. The theoretical product of each concentration of the substrate was calculated from the concentration of reducing sugars when the hydrolysis of 1% HPAC-pretreated softwood was nearly complete. The reactions were performed with 15 FPU cellulase g biomass^-1^ at 50 °C for 36 h.

## Data Availability

All data generated and analyzed in this study are included in this published article.

## Abbreviations

HPAC: Hydrogen peroxide acetic acid
CBHs: Cellobiohydrolases
EGs: Endoglucanases
Cel7A: Cellobiohydrolase
AnBgls: *Aspergillus niger* Betaglucosidases;
GtAA9: Gloeophyllum trabeum auxiliary activity family 9
Xyl: Xylanase
Bgl: Beta-glucosidase
