# Supplementary figures and images for: Hydrolysis pattern analysis of xylem tissues of woody plants pretreated with hydrogen peroxide and acetic acid: rapid saccharification of softwood for economical bioconversion
Source: Biotechnol Biofuels. 2021 Feb 6;14:37. doi: 10.1186/s13068-021-01889-y (PMC7866737; doi:10.1186/s13068-021-01889-y)

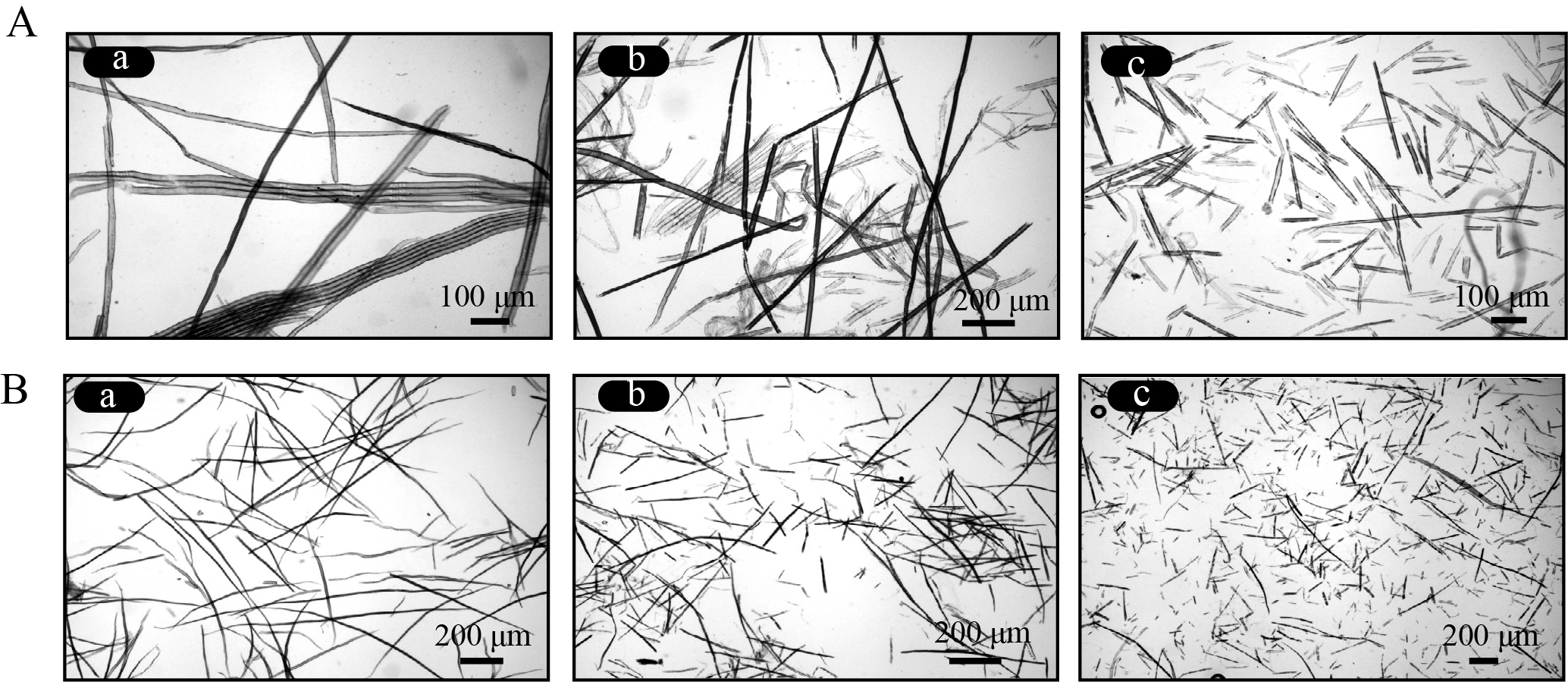

Supplement: Supplementary file 1 — Additional file 1: Figure S1. The fragmentations of HPAC-pretreated softwood and hardwood. (A) P. densiflora; (B) Q. acutissima. Tracheids or wood fibers from P. densiflora and Q. acutissima were hydrolyzed with 7.5 FPU cellulase (50 μL mL-1) g biomass-1 in 1 mL of 0.1 M citrate buffer (pH 5.0) at 50 °C. a, 0 h; b, 3 h; c, 6 h. [file 13068_2021_1889_MOESM1_ESM.jpg]

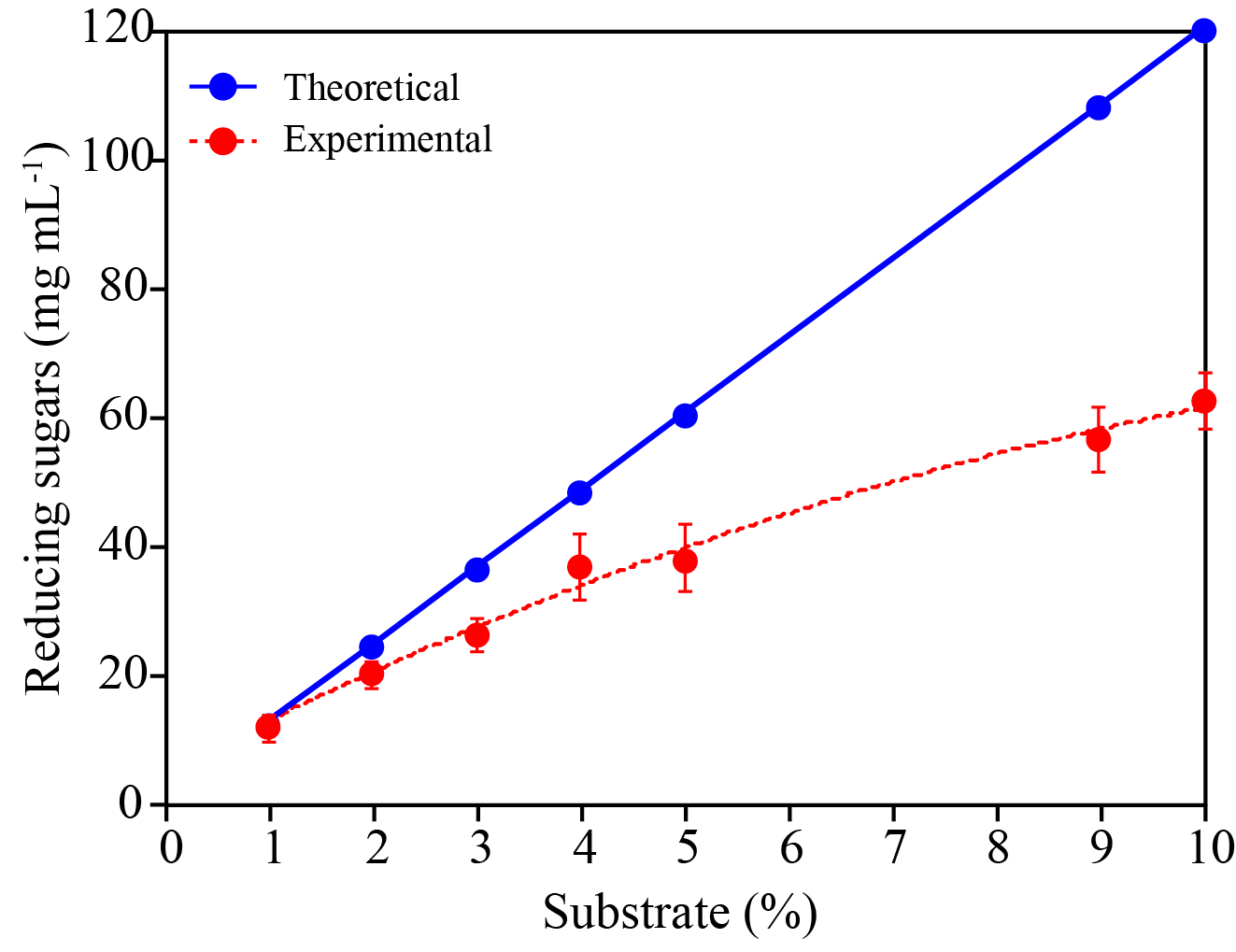

Supplement: Supplementary file 2 — Additional file 2: Figure S2. The comparison of end-product inhibition effect on various substrate concentrations of P. densiflora during enzymatic hydrolysis. The theoretical product of each concentration of the substrate was calculated from the concentration of reducing sugars when the hydrolysis of 1% HPAC-pretreated softwood was nearly complete. The reactions were performed with 15 FPU cellulase g biomass-1 at 50 °C for 36 h. [file 13068_2021_1889_MOESM2_ESM.jpg]
